# Supplementary material for: A Critical View of the Application of the APEX Software (Aqueous Photochemistry of Environmentally-Occurring Xenobiotics) to Predict Photoreaction Kinetics in Surface Freshwaters
Source: Molecules. 2019 Dec 18;25(1):9. doi: 10.3390/molecules25010009 (PMC7017383; doi:10.3390/molecules25010009)
Supplement: Supplementary file 1 [file molecules-25-00009-s001.zip › 3.2.4_gcc-4.4.0.zip!/3.2.4_gcc-4.4.0/3.2.4_gcc-4.4.0/doc/octave/html/interpreter/Object-Sizes.html]

Object Sizes - Untitled


Previous: User-defined Data Types,
Up: Data Types


---

### 3.3 Object Sizes

The following functions allow you to determine the size of a variable or
expression. These functions are defined for all objects. They return
−1 when the operation doesn't make sense. For example, Octave's
data structure type doesn't have rows or columns, so the `rows` and
`columns` functions return −1 for structure arguments.

— Built-in Function: **ndims** (a)  
> Returns the number of dimensions of array a.
> For any array, the result will always be larger than or equal to 2.
> Trailing singleton dimensions are not counted.

— Built-in Function: **columns** (a)  
> Return the number of columns of a.
>
>
>
> **See also:** size, numel, rows, length, isscalar, isvector, ismatrix.

— Built-in Function: **rows** (a)  
> Return the number of rows of a.
>
>
>
> **See also:** size, numel, columns, length, isscalar, isvector, ismatrix.

— Built-in Function: **numel** (a)  
> Returns the number of elements in the object a.
>
>
>
> **See also:** size.

— Built-in Function: **length** (a)  
> Return the `length' of the object a. For matrix objects, the
> length is the number of rows or columns, whichever is greater (this
> odd definition is used for compatibility with matlab).

— Built-in Function: **size** (a, n)  
> Return the number rows and columns of a.
>
> With one input argument and one output argument, the result is returned
> in a row vector. If there are multiple output arguments, the number of
> rows is assigned to the first, and the number of columns to the second,
> etc. For example,
>
> ```
>           size ([1, 2; 3, 4; 5, 6])
>                => [ 3, 2 ]
>           
>           [nr, nc] = size ([1, 2; 3, 4; 5, 6])
>                => nr = 3
>                => nc = 2
> ```
>
> If given a second argument, `size` will return the size of the
> corresponding dimension. For example
>
> ```
>           size ([1, 2; 3, 4; 5, 6], 2)
>                => 2
> ```
>
> returns the number of columns in the given matrix.
>
>
>
> **See also:** numel.

— Built-in Function: **isempty** (a)  
> Return 1 if a is an empty matrix (either the number of rows, or
> the number of columns, or both are zero). Otherwise, return 0.

— Built-in Function: **isnull** (x)  
> Return 1 if x is a special null matrix, string or single quoted string.
> Indexed assignment with such a value as right-hand side should delete array elements.
> This function should be used when overloading indexed assignment for user-defined
> classes instead of `isempty`, to distinguish the cases:
>
> `A(I) = []`: This should delete elements if `I` is nonempty. `X = []; A(I) = X`: This should give an error if `I` is nonempty.

— Built-in Function: **sizeof** (val)  
> Return the size of val in bytes

— Built-in Function: **size\_equal** (a, b, ...)  
> Return true if the dimensions of all arguments agree.
> Trailing singleton dimensions are ignored.
> Called with a single argument, size\_equal returns true.
>
>
>
> **See also:** size, numel.

— Built-in Function: **squeeze** (x)  
> Remove singleton dimensions from x and return the result.
> Note that for compatibility with matlab, all objects have
> a minimum of two dimensions and row vectors are left unchanged.
